# The Uses of Photodynamic Therapy Versus Anti-vascular Endothelial Growth Factor in the Management of Acute Central Serous Chorioretinopathy: A Systematic Review and Meta-Analysis

**DOI:** 10.7759/cureus.46100

**Published:** 2023-09-27

**Authors:** Hadeel Seraj, Mustafa Alhamoud, Mohammed S Khoshhal, Hassan Alhashim, Anas Alsaif, Amro Abukhashabah

**Affiliations:** 1 Department of Ophthalmology, King Abdulaziz University, Jeddah, SAU; 2 Department of Ophthalmology, King Fahd Hospital of the University, Dammam, SAU; 3 College of Medicine, Taibah University, Al-Madinah Al-Munawwarah, SAU; 4 Department of Ophthalmology, Imam Abdulrahman Bin Faisal University, Dammam, SAU; 5 College of Medicine, King Saud University, Riyadh, SAU; 6 Department of Ophthalmology, King Abdulaziz University, Rabigh, SAU

**Keywords:** central serous chorioretinopathy, acute cscr, intravitreal anti-vascular endothelial growth factor, photodynamic therapy, anti-vascular endothelial growth factor, sub-retinal fluids

## Abstract

Central serous chorioretinopathy (CSCR) is an idiopathic chorioretinal disease characterized by localized serous detachment of the neurosensory retina at the macula. To date, there is no high-quality evidence of recent updates on treating acute CSCR, focusing on photodynamic therapy (PDT) and anti-vascular endothelial growth factor (anti-VEGF). Hence, this review aims to conduct a comprehensive systematic review of the most recent therapeutic approaches for acute CSCR using the following electronic databases for a comprehensive and systematic literature review: MEDLINE, EMBASE, and Cochrane. In addition, we analyzed studies comparing PDT with placebo, anti-VEGF with placebo, or PDT with anti-VEGF in treating acute CSC eyes with no previous intervention. Seven studies were included, with a total of 292 eyes. The overall positive results were significantly higher among patients who received PDT compared to control groups (odds ratio [OR] = 7.96, 95% confidence interval [CI], 3.02 to 20.95, p < 0.001). The proportions of positive results were 81.0% and 97.1% among patients who received anti-VEGF and PDT, respectively, with no statistically significant differences between the groups. In addition, there were no significant differences between anti-VEGF and control groups. In contrast, PDT was significantly associated with lower recurrence odds than the control groups (OR = 0.12, 95% CI, 0.04 to 0.39, p = 0.042). According to our findings, PDT showed higher positive results than anti-VEGF in acute CSCR. In addition, PDT was significantly associated with a lower recurrence rate than the control group. However, the analysis needs to be confirmed and updated by large-scale, well-designed randomized clinical trials.

## Introduction and background

Pachychoroidopathy is an umbrella term that includes many conditions and one of them is central serous chorioretinopathy (CSCR). In pachychoroidopathy, there will be a thick choroid together with dilated hyperpermeable vessels, which results in focal Bruch’s membrane and retinal pigment epithelium (RPE) disruption leading to serous retinal detachment. CSCR is an idiopathic retinal disease characterized by localized serous detachment of the neurosensory retina at the macula due to leakage from the choriocapillaris secondary to hyperpermeable RPE spots [1,2]. CSCR predominantly affects young and middle-aged white adults with a male-to-female ratio of 3-10:1, and it is considered the fourth most common retinopathy just after age-related macular degeneration, diabetic macular edema, and branch retinal vein occlusion [1-3]. The exact pathophysiological mechanism is still an area of research. However, it is thought to involve the interaction of multiple factors like the dysfunction of the RPE layer along with the increased vascular permeability leading to hyperperfusion of the choroid and damage to the RPE barrier, causing the accumulation of fluid in the subretinal space to result in variable visual distortion [4,5].

Multiple risk factors have been associated with CSCR, including Caucasian race, stress and depression, pregnancy, alcohol, obstructive sleep apnea, and untreated hypertension [6,7]. However, the most important external risk factor for developing CSCR is corticosteroids [6]. Other associations include Cushing syndrome, systemic lupus erythematosus, organ transplantation, and end-stage renal disease [6,8]. The clinical presentation of CSCR ranges from reduced contrast sensitivity to acute visual loss, which includes a refractive error, mostly hyperopia, decreased vision, visual distortion like micropsia or metamorphopsia, and central scotoma [9]. The natural course of the disease is variable, which could be either acute CSCR in 80% of cases that is usually self-resolving in three to six months with good visual acuity, or chronic CSCR in about 15% of cases which lasts more than 12 months that might lead to macular degeneration, foveal atrophy with permanent long-term loss of vision [1,10]. Moreover, the risk of recurrence is impressively high, reaching up to 50% in untreated patients [11,12]. Diagnosing CSCR can be established by the fundoscopic examination which appears as a well-demarcated round dome shape detachment of the neurosensory retina at the macula [13]. Imaging studies such as optical coherence tomography (OCT), fluorescein angiography (FA), and indocyanine green angiography (ICGA) are helpful in ruling out the possible differential diagnosis, guiding the treatment strategy, and monitoring the disease progression [14]. The classic radiological finding of CSCR is the presence of subretinal fluid (SRF). Additionally, there will be intraretinal cystic changes in chronic cases visible in OCT imaging which is considered the first line of investigation [15]. Moreover, an ink blot pattern of leakage and smokestack leak appearance is appreciated by FA, while ICGA will demonstrate more details of choroidal vasculature as hypocyanescence in the early phase consistent with nonperfusion areas of the choriocapillaris and hypercyanescence in mid to late phases corresponding to the area of the leaks [14-16]. For the treatment, a watchful waiting approach is the standard of care for newly diagnosed acute CSCR, as most of them will spontaneously improve within three to six months [17]. However, one-eyed patients with CSCR or chronic and recurrent CSCR cases are usually indicated to initiate the treatment to prevent further damage to the neuroretina and irreversible visual loss [13]. Eliminating the risk factors is an essential component of the treatment plan, for example, discontinuing all forms of corticosteroids or offering an alternative therapy, if possible, for their medical condition [18]. Current treatment options for chronic or recurrent CSCR include medical treatment with low-dose aspirin and anti-corticosteroids, FA-guided laser photocoagulation, photodynamic therapy (PDT), and anti-vascular endothelial growth factor (anti-VEGF) therapy in case of choroidal neovascular membranes [19-22]. In acute CSCR, which has a reasonably high rate of spontaneous resolution, it is still debatable if it necessitates early intervention within three months of diagnosis. According to certain studies, some individuals may lose their vision following an initial spontaneous remission, and prompt interventions in acute CSCR may improve the final visual outcome and reduce the risk of recurrence [2,13,23,24], while other studies concluded that there were insignificant differences in final visual outcomes with early interventions in acute CSCR [25,26]. Certain complications were associated with the treatment of acute CSCR, such as permanent symptomatic scotomas corresponding to the laser photocoagulation scar sites; therefore, it is less likely to be used in acute CSCR [27]. While anti-VEGF and PDT have a favorable safety profile, they are commonly used in treating acute CSCR with controversial efficacy. Thus, it would be an area of discussion [28,29].

To this date, there is a lack of high-quality evidence of recent updates in treating acute CSCR, focusing on the role of PDT therapy and anti-VEGF. Therefore, the present review aims to systematically review the updated treatment of acute CSCR and investigate various aspects of the reported treatment strategies in addition to the meta-analysis to guide future clinical research and practice.

## Review

Methods

Literature Search

The present systematic review was conducted in accordance with the recommendations of the International Prospective Register of Systematic Reviews (PROSPERO), with the following ID number: CRD42022365688 [30]. The nature of the study did not require ethical approval. An electronic literature review was conducted on October 20, 2022, using MEDLINE, EMBASE, and Cochrane. The following keywords were used in our search: [(‘‘Chorioretinopathy’’ OR ‘‘CSC’’ OR ‘‘central serous chorioretinopathy’’) AND (‘‘anti-vascular endothelial growth factor’’ OR ‘‘ranibizumab’’ OR ‘‘bevacizumab’’ OR ‘‘aflibercept’’ OR ‘‘conbercept’’ OR ‘‘anti-VEGF’’) AND (‘‘photodynamic therapy’’ OR ‘‘verteporfin’’ OR ‘‘visudyne’’)]. To find any missed articles, we also searched Google Scholar. A search was conducted for additional articles in their reference lists once relevant articles had been identified. The selected articles were written in English and published without time limitations in October 2022. 

Study Selection

The following inclusion criteria were used: (1) studies for which raw numbers of patients underwent PDT or anti-VEGF; (2) studies of the following study designs (randomized clinical trials [RCTs], case-control, cross-sectional, prospective, or retrospective cohort studies); (3) adult population (18 years old and above); (4) studies that compared (i) PDT versus placebo; (ii) anti-VEGF versus placebo; or (iii) PDT versus anti-VEGF in the treatment of acute CSC eyes without previous intervention; (5) studies that were in English.

**Figure 1:**
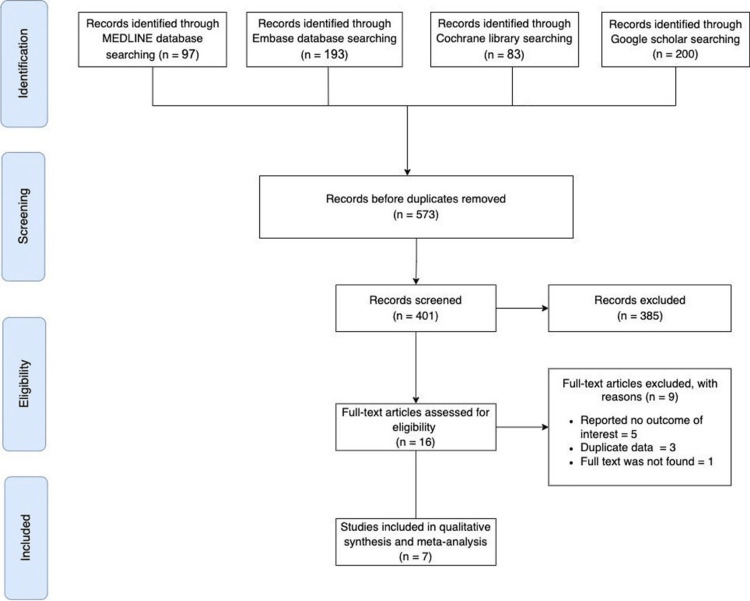
The PRISMA flowchart for searching and selecting the included studies

Our exclusion criteria were as follows: (1) study designs other than those included in the inclusion criteria (case report, meta-analysis/systematic review, economic analysis, animal study, cadaver study, narrative review, or editorial); (2) published in a language other than English; (3) reported no outcomes of interest. The following PRISMA chart demonstrates searching and selecting the included studies (Figure 1). 

Screening and Data Extraction

Four independent reviewers (MA, MK, HA, and AA) did a full-text screen of the included articles using the Rayyan application [31], where all potentially related articles were imported and retrieved, and duplicate studies were removed. Two authors (MK and MA) extracted the following data: author, year of publication, country, study design, number of patients in each group, age mean, gender, comorbidities, medication/intervention name, duration of intervention, dose, if there was previous therapy or not, number of affected eyes, recurrence of symptoms/complaint and its rate, follow-up period, overall complications, number of cases with complete resolution of the SRF, a gain of best-corrected vision (BCVA) measured by logMAR, and reduction in central foveal thickness (CFT) by micrometer documented by imaging. Two independent investigators (HM and HS) resolved disagreements and inconsistencies regarding data extraction. 

Quality Assessment 

Two independent reviewers (HA and AA) evaluated and assessed the methodological quality of the included articles by using a valid and previously reported assessment tool for both non-randomized and randomized studies [32]. It is composed of 27 items grouped into five main domains: external validity, confounding, bias, power, and reporting. The final answers were identical for both reviewers. The percentage of the maximum achievable score for each trial was used to express the total score. The quality of the study was labeled “good” if the score was more than or equal to 50%. 

Statistical Analysis

Statistical analysis was conducted using RStudio (R version 4.1.1). Categorical data were expressed as frequencies and percentages, and mean values of numerical outcomes were presented whenever applicable. The meta-analysis was carried out using a Mantel-Haenszel method, and the results were presented using odds ratios (ORs) and 95% confidence intervals (95% CIs) for complete resolution of the SRF, whereas relative risks and 95% CIs were used to analyze the difference in the risk of recurrence between groups. Pairwise comparisons with zero or complete (100%) events in both groups were not depicted in the forest plots. A heterogeneity assessment was carried out by conducting an I2 test, where a significant heterogeneity was deemed at I2 >50%. A fixed-effects model was used when the studies were similar enough that their results could be combined without introducing bias. Otherwise, a random-effects model was used to account for heterogeneity between studies. Assessment of publication bias and conducting a subgroup analysis were not possible in the current review owing to the small number of included studies.

Results

Characteristics of the Included Studies

In the current study, seven studies were eligible for inclusion in the systematic review and meta-analysis [23,24,33-37]. Table 1 demonstrates the characteristics of the included studies. One study was conducted in Europe (Turkey) [33], whereas the remaining six studies were carried out in Eastern Asian countries. Two RCTs were included [24,34], while three [35-37] and two studies [23,33] employed retrospective and prospective designs, respectively. We tried to minimize the impact of these differences by using a standardized methodology for data extraction and analysis. Three studies compared PDT vs. placebo/observation [24,35,37], three studies compared anti-VEGF vs. placebo/observation [23,33,36], and one study compared anti-VEGF vs. PDT [35]. Focusing on studies that used anti-VEGF (n=4), bevacizumab was exclusively used in two studies [33,35], ranibizumab in one study [23], and both medications were used in the remaining studies [36]. A half-dose PDT with verteporfin was employed in the studies with PDT arms [24,34,35,37]. The control groups consisted of giving a placebo (normal saline) in two studies [24,34] and observation in four studies [23,33,36,37], as demonstrated in Table 1. 

**Table 1:** Characteristics of the included studies PDT: photodynamic therapy; anti-VEGF: anti-vascular endothelial growth factor.

Author	Country	Design	Groups	No of patients (eyes)	Males/females	Interventions and doses	Follow-up
Kim et al. (2013) [23]	Korea	Prospective	Anti-VEGF	20 (20)	12/8	Ranibizumab (0.5 mg, single dose)	6 months
			Observation	20 (20)	10/10	Observation	6 months
Chan et al. (2008) [24]	Hong Kong	RCT	PDT	42 (42)	38/4	Half-dose PDT with verteporfin: 3 mg/m^2^ verteporfin, laser at 689 nm, light energy of 50 J/cm^2^	39 patients (12 months)
			Placebo	21 (21)	16/5	Placebo (normal saline 30 mL)	19 patients (12 months)
Aydin (2013) [33]	Turkey	Prospective	Anti-VEGF	13 (13)	12/1	Bevacizumab (0.08 mL, 2 mg)	6 months
			Observation	9 (9)	7/2	Observation	6 months
Wu et al. (2011) [34]	Hong Kong	RCT	PDT	24 (24)	21/3	Half-dose PDT with verteporfin: 3 mg/m^2^ verteporfin, laser at 689 nm, light energy of 50 J/cm^2^	12 months
			Placebo	10 (10)	8/2	Placebo (normal saline 30 mL)	12 months
Kim et al. (2015) [35]	Korea	Retrospective	Anti-VEGF	42 (42)	29/13	Bevacizumab (0.05 mL, 1.25 mg)	19.07 ± 6.80 months
			PDT	34 (34)	29/5	Half-fluence PDT with verteporfin: light energy (25 J/cm^2^), verteporfin (6 mg/m^2^) and half laser intensity (300 mW/cm^2^)	16.65 ± 4.99 months
Park et al. (2014) [36]	Korea	Retrospective	Anti-VEGF	21 (21)	14/7	6 eyes: bevacizumab (0.05 mL, 1.25 mg) and 15 eyes: ranibizumab (0.05 mL, 0.5 mg)	12 months
			Observation	15 (15)	11/4	Observation	12 months
Kim et al. (2014) [37]	Korea	Retrospective	PDT	10 (10)	6/4	Half-dose PDT with verteporfin: 3 mg/m^2^ verteporfin, laser at 689 nm, light energy of 50 J/cm^2^	12 months
			Observation	11 (11)	8/3	Observation	12 months

The included investigations recruited a total of 292 patients (292 eyes), of whom 221 patients were males (75.7%) and 71 females (24.3%). Anti-VEGF was administered to 96 patients (32.9%) and PDT to 110 patients (37.7%), whereas 86 patients were allocated to the control groups (29.5%, Table 1). 

Results of the Primary and Secondary Outcomes

In the meta-analysis, we included six studies that employed a placebo/observation because the remaining study, which compared anti-VEGF and PDT [35], had no possible investigations with a similar design (similar comparative groups). Therefore, we demonstrated below the meta-analysis results relying on six studies and a narrative overview of the study that compared anti-VEGF and PDT [35].

**Figure 2:**
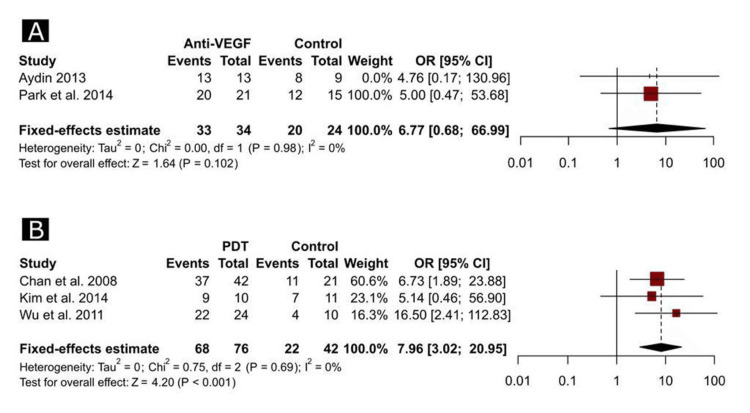
Forest plots depicting the odds ratio of complete resolution of the subretinal fluid between anti-VEGF and control groups (A) and between PDT and control groups (B). Refs. [24,33,34,36,37]. PDT, photodynamic therapy; VEGF, vascular endothelial growth factor; OR, odds ratio; CI, confidence interval.

The overall favorable results (defined as complete resolution of the SRF) were not significantly different between anti-VEGF and control groups (Figure 2A); however, the OR of favorable outcomes was significantly higher among patients who received PDT compared to control groups (OR = 7.96, 95% CI, 3.02 to 20.95, p < 0.001, Figure 2B). In the study that directly compared the active interventions [35], the proportions of positive results were 81.0% and 97.1% among patients who received anti-VEGF and PDT, respectively, with no statistically significant differences between the groups. 

**Figure 3:**
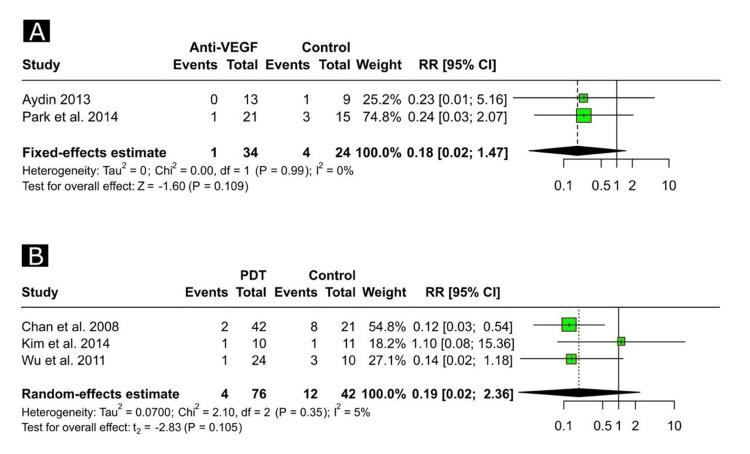
Forest plots depicting the risk ratio of recurrence between anti-VEGF and control groups (A) and between PDT and control groups (B). Refs. [24,33,34,36,37]. PDT, photodynamic therapy; VEGF, vascular endothelial growth factor; CI, confidence interval; RR, risk ratio.

In the study of Kim et al. [35], the recurrence rate was significantly higher in the anti-VEGF group than in the PDT group (19.0% vs. 2.9%, p = 0.037). Based on the outcomes of the meta-analysis, there were no significant differences between anti-VEGF and control groups (Figure 3A), whereas PDT was significantly associated with lower odds of recurrence compared to the control groups (OR = 0.12, 95% CI, 0.04 to 0.39, p = 0.042, Figure 3B). In addition, the meta-analytical approaches showed no significant heterogeneity across all the pairwise comparisons (I2 = 0% for all comparisons in Figure 2 and Figure 3). 

Notably, pairwise comparisons of one study [23] had zero events in both groups in the recurrence outcome and complete events (100%) in both groups in the rate of complete resolution; therefore, the results of such a study were not reported in the respective forest plots (Figure 2 and Figure 3).

For other outcomes, we could not analyze the continuous variables (mean BCVA gain and the mean reduction in CFT) because these outcomes were reported as mean values without measures of variance (standard deviation, standard errors, etc.). Nevertheless, these results are demonstrated in Table 2. All the studies reported no possible complications; hence, no additional treatments were adopted. 

**Table 2:** Results of individual studies regarding the mean BCVA gain and CFT reduction across different interventions and control groups. PDT: photodynamic therapy; anti-VEGF: anti-vascular endothelial growth factor; BCVA: best-corrected vision; CFT, central foveal thickness. ^*^Gain of BCVA measured by logMAR. ^¥^Reduction in CFT by micrometer documented by imaging.

Author	Mean BCVA gain*			Mean reduction in CFT^¥^		
	Anti-VEGF	PDT	Control	Anti-VEGF	PDT	Control
Kim et al. (2013) [23]	0.20	NA	0.13	240	NA	205
Chan et al. (2008) [24]	NA	0.21	0.06	NA	296	174
Aydin (2013) [33]	0.34	NA	0.42	216	NA	305
Wu et al. (2011) [34]	NA	0.18	0.01	NA	326	156
Kim et al. (2015) [35]	0.04	0.11	NA	77.61	84.71	NA
Park et al. (2014) [36]	0.16	NA	0.12	239	NA	187
Kim et al. (2014) [37]	NA	0.20	0.07	NA	270	164

Quality Assessment

Two impartial observers (MK, HA) used a previously defined quality rating system for randomized and non-randomized studies to assess the effectiveness of the included clinical trials [32]. Five subscales were composed of the following 27 items: reporting (10 items), external validity (three items), bias (seven items), confounding (six items), and power (one item). A judgment was taken after discussing any discrepancies between the two observers' qualitative evaluations. Scores were expressed as a percentage of the highest possible score for each trial, and a score of 50% or higher is considered high quality (Table 3). 

**Table 3:** An assessment of the quality of the included studies.

Author (year)	Quality score component					Score	
	I	II	III	IV	V	Overall	Percentage
Kim et al. (2013) [23]	9	3	5	4	0	21	65.62
Chan et al. (2008) [24]	9	3	7	5	2	26	81.25
Aydin (2013) [33]	8	0	5	2	0	15	46.88
Wu et al. (2011) [34]	7	3	6	4	0	20	62.5
Kim et al. (2015) [35]	8	3	5	3	0	19	59.37
Park et al. (2014) [36]	9	3	5	3	0	20	62.5
Kim et al. (2014) [37]	9	3	5	3	0	20	62.5

Discussion

It is a well-known fact that acute CSC has a self-limiting nature. Thus, there is a consensus that watchful waiting for at least three months is the current standard of care for cases of acute CSC before considering other treatment modalities. However, those who need rapid recovery and desire to regain their vision before spontaneous resolution can benefit from other treatment modalities like anti-VEGF and PDT. As there is limited data on the role of anti-VEGF and PDT in treating acute CSC, this review aims to provide an update and to compare PDT, anti-VEGF, and observation in the management of acute CSC.

This systematic review included seven articles with 292 patients (292 eyes) diagnosed with acute CSCR. All studied patients were managed by PDT, anti-VEGF (bevacizumab, ranibizumab), placebo, or observation. Currently, there are several studies regarding the management of acute CSC; those studies are mostly non-RCT.

CSC may cause SRF, mainly from exudative CNV activity. Hence, anti-VEGF treatment might be an effective therapeutic option [38]. Anti-VEGF antibodies have recently been employed in the management of acute CSC. Anti-VEGF agents are known to affect choroidal circulation by reducing choroidal hyperpermeability and thickness through the suppression of nitric oxide production and constriction of pachychoroid vessel. Therefore, intravitreal injection of bevacizumab has been shown to be less effective in treating chronic CSCR patients without CNV than in those with CNV [38]. After being injected intravitreally, bevacizumab, a recombinant humanized full-length monoclonal antibody of VEGF, can pass through the retina and reach the RPE, choroid, and outer segments of photoreceptors. However, ranibizumab, due to its smaller molecular size and higher binding ability to VEGF, might penetrate the retina more than bevacizumab. This paper covered two non-RCT studies to compare the difference in complete resolution of the SRF and the risk of recurrence in patients who underwent anti-VEGF therapy compared to those managed by observation. According to meta-analysis, acute CSC patients who were managed with anti-VEGF therapy showed no significant difference in complete resolution of the SRF and increased risk of recurrence compared to patients managed by observation only. However, anti-VEGF has the advantage of producing rapid improvement in neurosensory retinal detachment and visual acuity. Although anti-VEGF produces rapid improvement, the result of follow-up with patients who underwent anti-VEGF or observation is similar. Therefore, the use of anti-VEGF might be considered for patients in certain occupations or conditions who seek rapid improvement in visual function [23,33,36]. In addition, Kim et al. reported that patients treated with anti-VEGF have a higher risk of recurrence, which is inconsistent with the meta-analysis that showed no significant difference [35]. However, the short-term visual outcomes with anti-VEGF were observed to be better in patients with a thinner choroid and smaller pachychoroid vessel after bevacizumab injection for chronic CSC with CNV. Therefore, conventional treatments like focal laser and PDT may be more appropriate for chronic CSC patients without CNV [38]. Song et al. further emphasized that smaller pachychoroid arteries and thinner choroidal thickness at presentation in CNV patients were related to improved short-term visual results [38].

PDT is a successful treatment for acute CSC, and research has turned to employ half-dose verteporfin to lessen its side effects. Since PDT works by reducing choroidal hyperpermeability, half a dose of PDT is sufficient to produce that effect. Two RCT studies and one non-RCT study that examine the difference in complete resolution of the SRF and risk of recurrence in patients who underwent half-dose PDT with verteporfin therapy versus placebo/observation were included in this article. According to meta-analysis, there is a significant difference in the complete resolution of the SRF and a lower risk of recurrence in patients managed by PDT compared to patients treated by observation/placebo [24,34,37]. Two RCTs used bevacizumab and ranibizumab intravitreal injections to treat acute CSC. Meta-analysis showed that patients treated with anti-VEGF have significantly more BVCA gain and reduction in CFT than those managed by observation. However, there is a need for more objective measures of visual function changes, for example, contrast sensitivity and retinal sensitivity [23,36]. Complications of therapy, such as RPE atrophy and macular ischemia, were not reported in all the included articles.

Limitations and future recommendations

This systematic review and meta-analysis is the most updated review comparing PDT, anti-VEGF, and observation in treating acute CSC. Nevertheless, we acknowledge that there are several limitations in our study. First, most of our results were based on retrospective cohort studies with relatively small sample sizes, and only two included studies were randomized clinical trials. Secondly, statistical analyses of the mean BCVA gain and the mean reduction in CFT were not possible as measures of variance (standard deviation, standard errors, etc.) needed to be included in the outcomes of these variables. In addition, one of the included studies had a score of less than 50% in the quality assessment; thus, this could be a potential source of bias. Finally, most of the included articles originated from South Korea and China; thus, the generalizability of the results to non-Asian regions can be difficult.

Acute CSC should be treated promptly to reach better gain. Although anti-VEGF showed more rapid absorption of SRF, PDT stands as the desirable treatment for acute CSC as it improves function and anatomy upon observation. Future studies should expand the number of patients and follow-up duration. In addition, other measures of visual function should be added, such as contrast sensitivity, microperimetry, and electrophysiological measurements, to help decide the preferred treatment time and patient prognosis.

## Conclusions

The present study is the most updated systematic review focused on whether PDT or anti-VEGF therapy is more effective for acute CSCR. Our findings showed no significant difference in the complete resolution of SRF between the anti-VEGF and control groups. Compared with anti-VEGF, PDT showed higher positive results in acute CSCR. Furthermore, PDT was significantly associated with a lower recurrence rate compared to the control group. Although these findings are encouraging, it is essential to note that the included studies have inherent limitations and that conclusions drawn from the pooled results should be interpreted cautiously. This analysis needs to be confirmed and updated by large-scale, well-designed RCTs with extensive follow-up.

## References

[1] Salmon JF (2019). Kanski’s Clinical Ophthalmology, 9th ed.

[2] van Rijssen TJ, van Dijk EH, Yzer S (2019). Central serous chorioretinopathy: Towards an evidence-based treatment guideline. Prog Retin Eye Res.

[3] Desai UR, Alhalel AA, Campen TJ, Schiffman RM, Edwards PA, Jacobsen GR (2003). Central serous chorioretinopathy in African Americans. J Natl Med Assoc.

[4] Yoshioka H, Katsume Y (1982). Experimental central serous chorioretinopathy. III: Ultrastructural findings. Jpn J Ophthalmol.

[5] Nicholson B, Noble J, Forooghian F, Meyerle C (2013). Central serous chorioretinopathy: Update on pathophysiology and treatment. Surv Ophthalmol.

[6] Haimovici R, Koh S, Gagnon DR, Lehrfeld T, Wellik S (2004). Risk factors for central serous chorioretinopathy: A case-control study. Ophthalmology.

[7] Tittl MK, Spaide RF, Wong D (1999). Systemic findings associated with central serous chorioretinopathy. Am J Ophthalmol.

[8] Ulbig MR, Riordan-Eva P, Holz FG, Rees HC, Hamilton PA (1993). Membranoproliferative glomerulonephritis type II associated with central serous retinopathy. Am J Ophthalmol.

[9] Liew G, Quin G, Gillies M, Fraser-Bell S (2013). Central serous chorioretinopathy: A review of epidemiology and pathophysiology. Clin Exp Ophthalmol.

[10] Mrejen S, Balaratnasingam C, Kaden TR (2019). Long-term visual outcomes and causes of vision loss in chronic central serous chorioretinopathy. Ophthalmology.

[11] Berger L, Bühler V, Yzer S (2021). Central serous chorioretinopathy - An overview. Klin Monbl Augenheilkd.

[12] Gilbert CM, Owens SL, Smith PD, Fine SL (1984). Long-term follow-up of central serous chorioretinopathy. Br J Ophthalmol.

[13] Abouammoh MA (2015). Advances in the treatment of central serous chorioretinopathy. Saudi J Ophthalmol.

[14] Borrelli E, Sarraf D, Freund KB, Sadda SR (2018). OCT angiography and evaluation of the choroid and choroidal vascular disorders. Prog Retin Eye Res.

[15] Kim HC, Cho WB, Chung H (2012). Morphologic changes in acute central serous chorioretinopathy using spectral domain optical coherence tomography. Korean J Ophthalmol.

[16] Yamada K, Hayasaka S, Setogawa T (1992). Fluorescein-angiographic patterns in patients with central serous chorioretinopathy at the initial visit. Ophthalmologica.

[17] Yannuzzi LA (1986). Type A behavior and central serous chorioretinopathy. Trans Am Ophthalmol Soc.

[18] Loo RH, Scott IU, Flynn HW Jr (2002). Factors associated with reduced visual acuity during long-term follow-up of patients with idiopathic central serous chorioretinopathy. Retina.

[19] Caccavale A, Romanazzi F, Imparato M, Negri A, Morano A, Ferentini F (2010). Low-dose aspirin as treatment for central serous chorioretinopathy. Clin Ophthalmol.

[20] Quin G, Liew G, Ho IV, Gillies M, Fraser-Bell S (2013). Diagnosis and interventions for central serous chorioretinopathy: Review and update. Clin Exp Ophthalmol.

[21] Ruiz-Moreno JM, Lugo FL, Armadá F (2010). Photodynamic therapy for chronic central serous chorioretinopathy. Acta Ophthalmol.

[22] Konstantinidis L, Mantel I, Zografos L, Ambresin A (2010). Intravitreal ranibizumab in the treatment of choroidal neovascularization associated with idiopathic central serous chorioretinopathy. Eur J Ophthalmol.

[23] Kim M, Lee SC, Lee SJ (2013). Intravitreal ranibizumab for acute central serous chorioretinopathy. Ophthalmologica.

[24] Chan WM, Lai TY, Lai RY, Liu DT, Lam DS (2008). Half-dose verteporfin photodynamic therapy for acute central serous chorioretinopathy: One-year results of a randomized controlled trial. Ophthalmology.

[25] Chung YR, Seo EJ, Lew HM, Lee KH (2013). Lack of positive effect of intravitreal bevacizumab in central serous chorioretinopathy: Meta-analysis and review. Eye (Lond).

[26] Chrapek O, Jirkova B, Kandrnal V, Rehak J, Sin M (2015). Treatment of central serous chorioretinopathy with beta-blocker metipranolol. Biomed Pap Med Fac Univ Palacky Olomouc Czech Repub.

[27] Ross A, Ross AH, Mohamed Q (2011). Review and update of central serous chorioretinopathy. Curr Opin Ophthalmol.

[28] Hagen S, Ansari-Shahrezaei S, Smretschnig E, Glittenberg C, Krebs I, Graf A, Binder S (2013). The effect of photodynamic therapy on macular sensitivity in eyes with acute central serous chorioretinopathy. Graefes Arch Clin Exp Ophthalmol.

[29] Seong HK, Bae JH, Kim ES, Han JR, Nam WH, Kim HK (2009). Intravitreal bevacizumab to treat acute central serous chorioretinopathy: Short-term effect. Ophthalmologica.

[30] Shamseer L, Moher D, Clarke M (2015). Preferred reporting items for systematic review and meta-analysis protocols (PRISMA-P) 2015: Elaboration and explanation. BMJ.

[31] Ouzzani M, Hammady H, Fedorowicz Z, Elmagarmid A (2016). Rayyan-a web and mobile app for systematic reviews. Syst Rev.

[32] Downs SH, Black N (1998). The feasibility of creating a checklist for the assessment of the methodological quality both of randomised and non-randomised studies of health care interventions. J Epidemiol Community Health.

[33] Aydin E (2013). The efficacy of intravitreal bevacizumab for acute central serous chorioretinopathy. J Ocul Pharmacol Ther.

[34] Wu ZH, Lai RY, Yip YW, Chan WM, Lam DS, Lai TY (2011). Improvement in multifocal electroretinography after half-dose verteporfin photodynamic therapy for central serous chorioretinopathy: A randomized placebo-controlled trial. Retina.

[35] Kim DY, Joe SG, Yang HS, Lee JY, Kim JG, Yoon YH (2015). Subfoveal choroidal thickness changes in treated idiopathic central serous chorioretinopathy and their association with recurrence. Retina.

[36] Park SU, Lee SJ, Kim M (2014). Intravitreal anti-vascular endothelial growth factor versus observation in acute central serous chorioretinopathy: One-year results. Korean J Ophthalmol.

[37] Kim KS, Lee WK, Lee SB (2014). Half-dose photodynamic therapy targeting the leakage point on the fluorescein angiography in acute central serous chorioretinopathy: A pilot study. Am J Ophthalmol.

[38] Song YY, Yu HY, Baek SK, Lee YH, Lee MW (2021). Short-term effect of anti-VEGF for chronic central serous chorioretinopathy according to the presence of choroidal neovascularization using optical coherence tomography angiography. PLoS One.

